# Posterior urethral polyp in a male child: a rare case report

**DOI:** 10.1093/omcr/omac131

**Published:** 2022-12-16

**Authors:** Ali Razzok, Muhamad Sinan Muhamad, Ali Ismaeel, Khaled Alyousef, Majdy Oukan

**Affiliations:** Division of Urology, Department of Surgery, Tishreen University Hospital, Latakia, Syria; Division of Urology, Department of Surgery, Tishreen University Hospital, Latakia, Syria; Division of Urology, Department of Surgery, Tishreen University Hospital, Latakia, Syria; Division of Urology, Department of Surgery, Tishreen University Hospital, Latakia, Syria; Division of Urology, Department of Surgery, Tishreen University Hospital, Latakia, Syria

## Abstract

Urethral polyps are one of the rare deformities of the urethra. In most cases, the urethral polyps would not be considered in the differential diagnosis process by a huge number of physicians, mainly owing to the rarity of documented cases in the medical literature and because of the wide variety of unspecified symptoms the urethral polyp might demonstrate. Urethral polyps are more common in males than in females, and they are usually diagnosed at an early age. Treatment options include transurethral resection, endoscopic suprapubic approach and open surgery. The disease prognosis is excellent as it does not usually recur after being completely removed and the risk of malignant transformation is very low. We are going to report a case of a 3-month-old boy who presented with bilateral vesicoureteral reflux and hydronephrosis, which revealed the presence of a large posterior urethral polyp.

## INTRODUCTION

Urethral polyps are irregular growths that occur in the urethra. They are rare, and they are more common in males than in females. Posterior urethral polyps (PUPs) are more frequent than anterior urethral polyps, which are very rare. John Hunter is credited with the first documented case of urethral polyps in the literature, which he described in an ox in 1763 [1], whereas Thompson was the first to report this case in a patient in 1855 [2]. In 1899, Neuberger diagnosed the first urethral polyp using an endoscope [3]. Thus far, there has not been a plethora of documented cases about urethral polyps in the medical literature.

PUPs are benign irregular growths in the posterior urethra. Although the lesion is uncommon, it is considered as an important cause of bladder outlet obstruction. Because of its obstructive qualities, there is a wide range of clinical symptoms that include dysuria, recurrent urinary infections, hematuria and urinary retention. Infrequently, urethral polyps may be associated with vesicoureteral reflux (VUR) and hydronephrosis and this is what we are going to report in our case.

## CASE PRESENTATION

**Figure 1:**
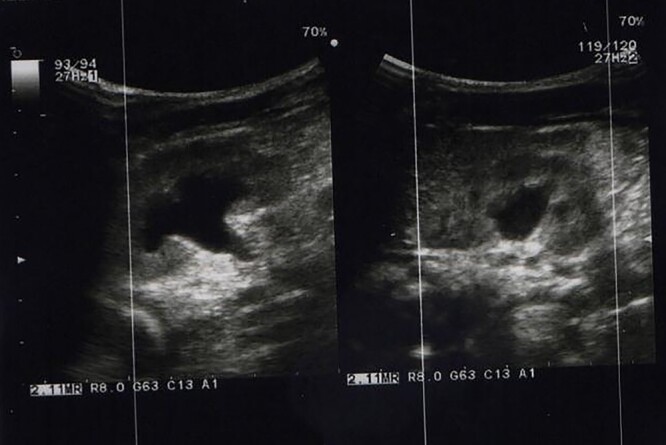
Ultra sound imaging showed the hydroureteronephrosis on both sides.

**Figure 2:**
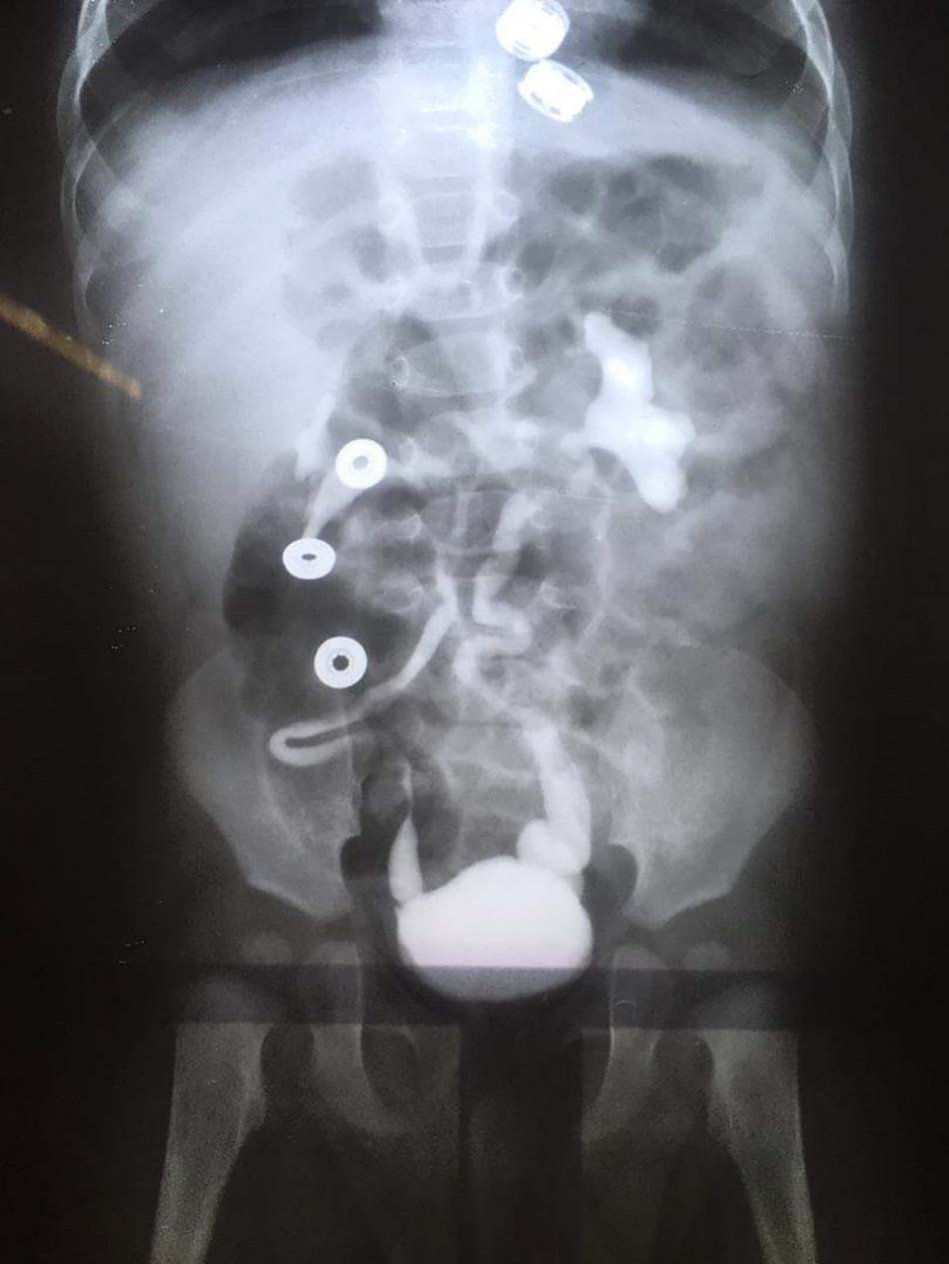
VCUG imaging was done. VUR was detected on both sides.

**Figure 3:**
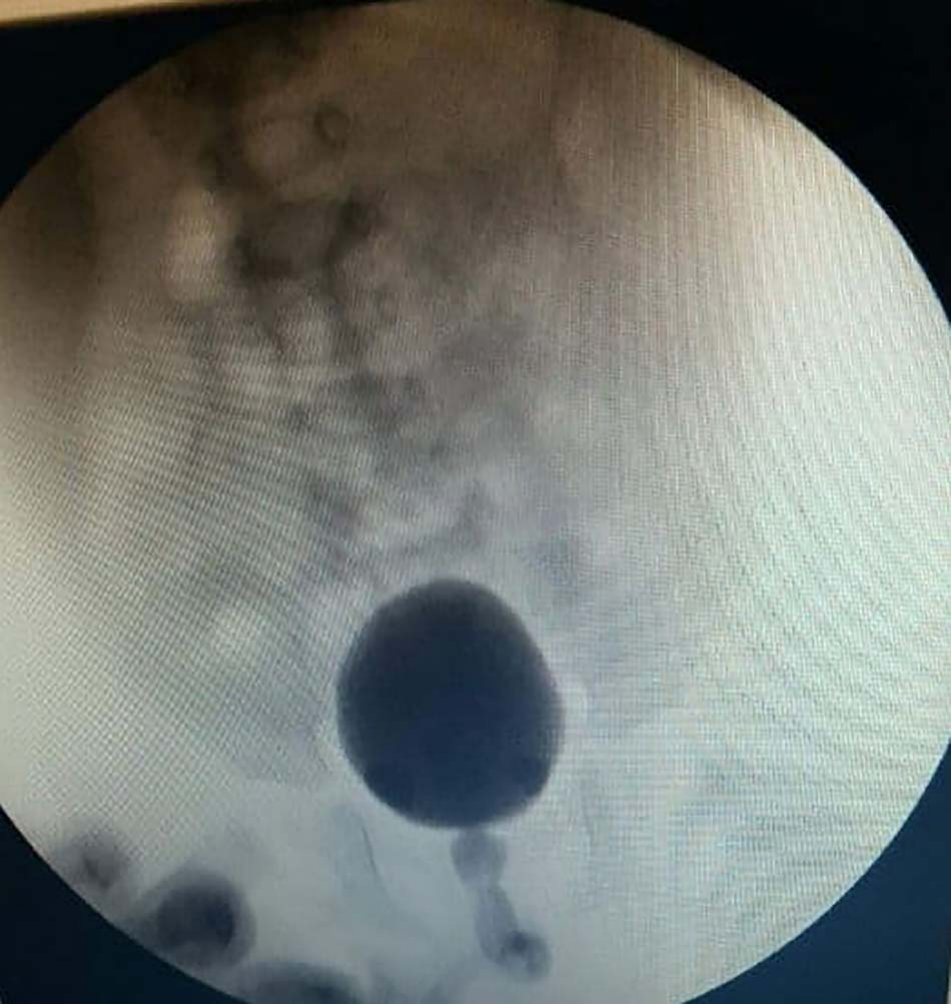
VCUG shows a dilated urethra with a filling defect in the prostatic urethra.

**Figure 4:**
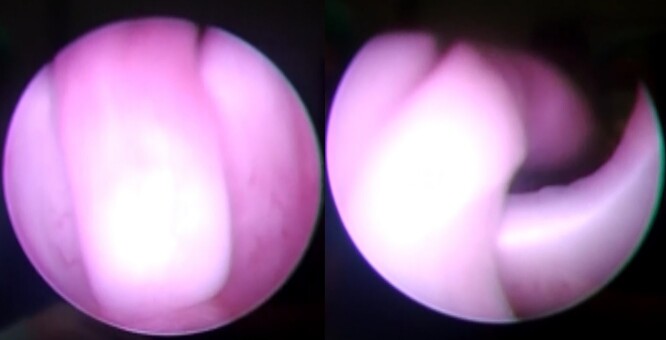
Cystourethroscopy revealed a polypoid mass arising from the verumontanum and extending into the bladder.

**Figure 5:**
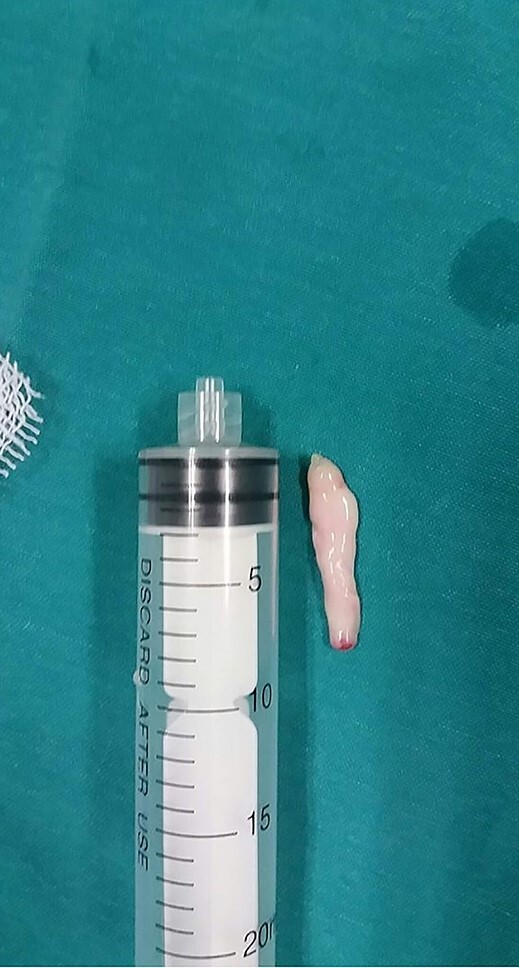
The removed urethral polyp.

A 3-month-old boy presented with a history of unexplained excessive crying episodes, mostly occur during micturition. On hospital admission, physical examination was normal. Laboratory studies including blood and urine tests were normal, as well. The patient underwent abdominal and pelvic ultrasound, which showed bilateral hydroureteronephrosis involving the dilation of the entire ureters (Fig. 1). Voiding cystourethrography (VCUG) was performed that revealed a moderate VUR on both sides (Fig. 2). In addition, a dilation with a filling defect in the prostatic urethra was detected (Fig. 3). Cystourethroscopy unfolded a urethral polyp 7.5^*^0.5^*^0.5 cm in size. The polyp pedicle was attached to the verumontanum ile the polyp extended into the bladder (Fig. 4). We also detected a small diverticulum on the posterior wall of the bladder. Transurethral resection of the lesion was done using endoscopic scissor, and the resescted mass was removed out of the urethra by urethral foreign body forceps (Fig. 5). Histopathology examination showed that the lesion is a polyp lined with squamous epithelia and covered with a smooth muscle layer, with no signs of malignancy.

An indwelling urinary catheter was used after surgery. 24 h later the boy was discharged home in perfect condition. The boy’s parents reported later that the micturition-related crying became significantly less recurrent.

**Figure 6:**
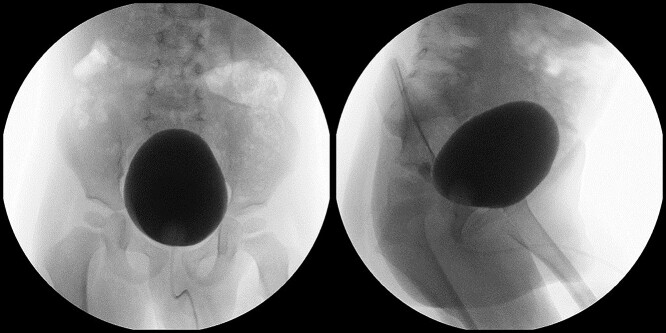
VCUG was repeated 1 year after removing the polyp. No filling defect, hydronephrosis nor VUR were detected.

1 year following the surgery, we conducted a new ultrasound and VCUG imaging which affirmed that the VUR and the hydronephrosis fully retreated on both sides (Fig. 6). In addition, a follow-up cystoscopy was performed to confirm that there was no recurrence of the polyp.

## DISCUSSION

Urethral polyps are uncommon benign polypoid masses of the urethra, and they typically manifest as single tumors rather than multiple separate masses. Until 2004, 181 cases of urethral polyps have been reported [4]. Since then, only a few cases have been found and described in the medical literature. Diagnosing urethral polyps might be difficult and carries a challenging differential diagnosis for clinical practitioners and physicians, because they are infrequent and they could present in a large variety of unspecified symptoms. Urethral polyps are notably more prevalent in males than in females [5]. In males, polyps that arise from the posterior urethra are significantly more frequent than anterior urethral polyps, which are rarely described in the literature [6]. This entity is extremely rare in females, and it could be located in any part of the urethra without any specific common location [7].

The pathogenesis of this condition is still poorly understood and controversial. However, congenital, irritating, septic, traumatic and obstructive factors have been discussed and proposed as potential causes for urethral polyps. Moore and Kuppusami suggest that maternal estrogen levels during gestation could cause secondary epithelial changes which in turn might develop urethral polyps [8]. Because the higher portion of cases diagnosed around birth is seen in healthy newborns and most cases are diagnosed in the first decade of life, the congenital theory is considered the most reliable in explaining urethral polyp’s origin [9].

Urethral polyps are typically asymptomatic [10]. Nevertheless, the clinical manifestations are widely diverse and mainly associated with age. In early infancy, intensive crying especially while urinating is the most prominent symptom, whereas older patients often present with hematuria, dysuria, recurrent urinary tract infection and urinary retention [11]. In regard with the obstructive characteristics of the lesion, repetitive urine retention and stasis could result in developing VUR and hydronephrosis. The polyp may protrude from the urethral meatus, mainly in females.

Although ultrasound imaging is important in the diagnosing process, it is not diagnostic and cannot detect the lesion successfully in most cases. VCUG assists in establishing diagnosis by showing a filling defect in the urethra and it also reveals accompanying comorbidities like VUR. The presence of urethral polyp is detected mainly by endoscopy and histologically confirmed by biopsy [11].

The standard treatment for urethral polyps is endoscopic transurethral resection by either cold knife, scissor, electrocoagulation or laser [12]. The resected polyp should be fully removed and taken out of the urethra, either by foreign body forceps or a stone basket. For polyps with larger sizes, turning the polyp into fragments and the removal of them all using a transurethral approach is the treatment of choice. Open approach surgery is indicated when the transurethral resection or the endoscopic suprapubic approach cannot be done. The urethral polyps prognosis is excellent, as the symptoms usually fully subside after proper management and the tendency of recurrence or malignant degeneration is seldom [13].

The follow-up by doing ultrasonography and VCUG should be done after 1 year. This is what we did in our case making sure that there was no recurrence and no VUR or any other abnormalities.

## CONCLUSION

Urethral polyps are always considered a distinctive medical case because they are rarely discussed and described in the literature and they could be demonstrated with a wide spectrum of symptoms. As it stands, they should always be considered as an important differential diagnosis for patients with voiding dysfunction or lower urinary tract symptoms, especially in the first decade of life.
